# Human Organ Culture: Updating the Approach to Bridge the Gap from *In Vitro* to *In Vivo* in Inflammation, Cancer, and Stem Cell Biology

**DOI:** 10.3389/fmed.2017.00148

**Published:** 2017-09-11

**Authors:** Rafia S. Al-Lamki, John R. Bradley, Jordan S. Pober

**Affiliations:** ^1^Department of Medicine, NIHR Cambridge Biomedical Research Centre, University of Cambridge, Cambridge, United Kingdom; ^2^Department of Immunobiology, Yale University School of Medicine, New Haven, CT, United States

**Keywords:** human, organ culture, inflammation, cancer, stem cells

## Abstract

Human studies, critical for developing new diagnostics and therapeutics, are limited by ethical and logistical issues, and preclinical animal studies are often poor predictors of human responses. Standard human cell cultures can address some of these concerns but the absence of the normal tissue microenvironment can alter cellular responses. Three-dimensional cultures that position cells on synthetic matrices, or organoid or organ-on-a-chip cultures, in which different cell spontaneously organize contacts with other cells and natural matrix only partly overcome this limitation. Here, we review how human organ cultures (HOCs) can more faithfully preserve *in vivo* tissue architecture and can better represent disease-associated changes. We will specifically describe how HOCs can be combined with both traditional and more modern morphological techniques to reveal how anatomic location can alter cellular responses at a molecular level and permit comparisons among different cells and different cell types within the same tissue. Examples are provided involving use of HOCs to study inflammation, cancer, and stem cell biology.

## Introduction

Preclinical animal studies have had only limited success in predicting human physiology, pathology, and therapeutic responses. Traditional human cell cultures, which are often used to account for species differences are also limited in their representation of *in vivo* responses due to lack of an appropriate microenvironmental context of the responding cell types. Newer culture approaches, such as three-dimensional (3D) cultures, organoids, or organs-on-a-chip have attempted to better replicate the tissue microenvironment, but have been only partly successful. In this review, we will illustrate how human organ cultures (HOCs) offer a simple approach that may better address these issues. To do so, we have chosen illustrative examples from the literature from the past 30 years that have been used to gain insights into inflammatory process and diseases, cancer, and stem cell biology. More recent examples have illustrated how newer morphological approaches, such as *in situ* proximity ligation assay, can be applied to HOCs to reveal greater details in specific biological responses.

## Methodology of HOC

### The Method Described Here Reflects Our Approach for Optimizing HOC

**Figure 1:**
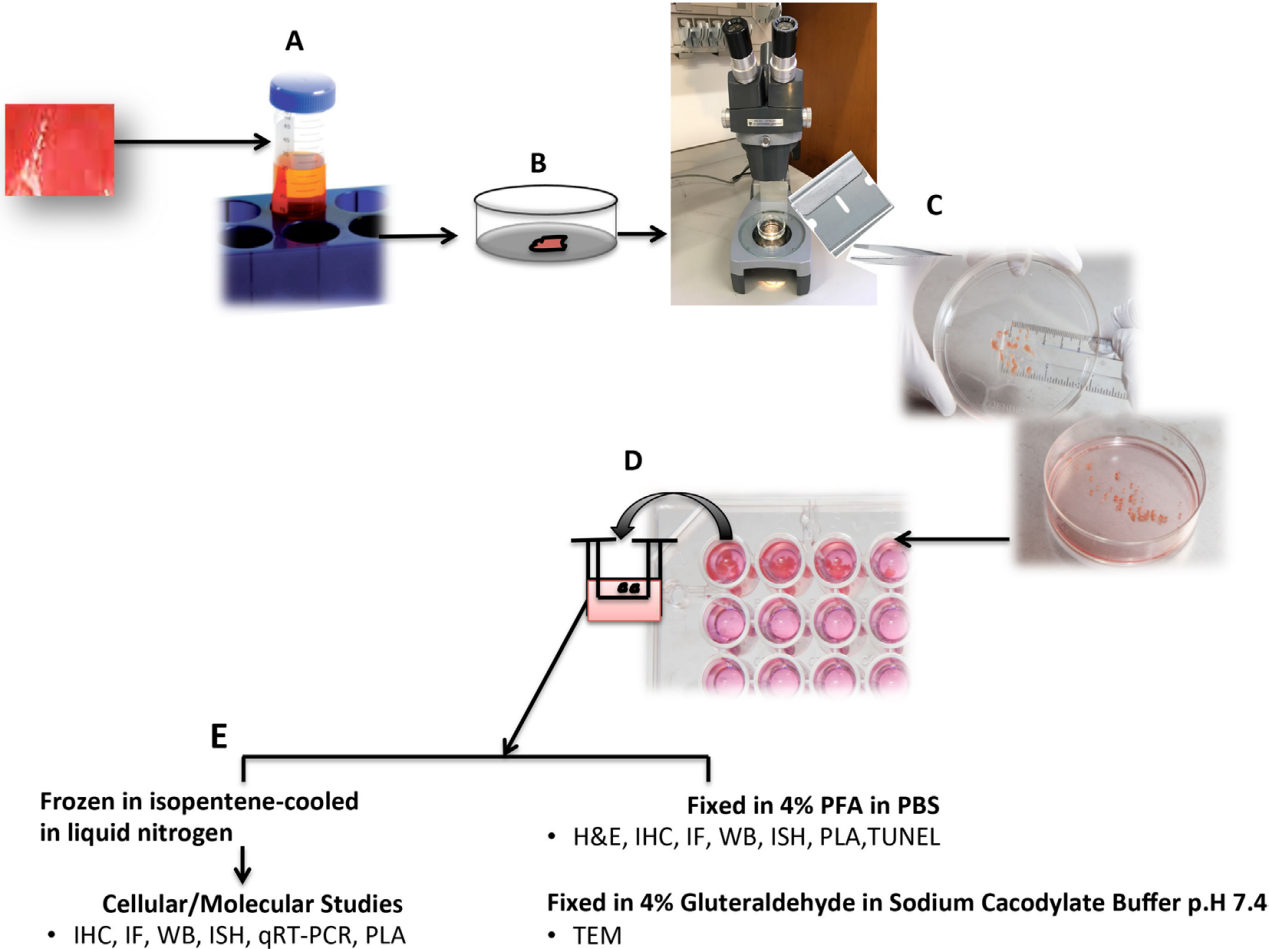
Schematic diagram of human organ culture (HOC) setup. Tissue is immersed in a sterile universal container with tissue culture medium (M199 supplemented with 5% heat-inactivated fetal calf serum, 100 U/ml penicillin/streptomycin, 2.2 mM l-glutamine) **(A)**. Tissue is rinsed in sterile phosphate-buffered saline (PBS) to remove excess blood **(B)**. Tissue is dissected under a dissecting microscope using two sterilized carbon steel razor blades single-edged stuck together creating a gap of <1 mm^3^ **(C)**. Tissue fragments are immersed in sterile 96-well plate containing 200 µl medium and sterile culture insert, treated as indicated, or left untreated and maintained in a 37°C incubator (95% air, 5% CO_2_) for the indicated time periods (usually less than 24 h) **(D)**. Upon harvest, HOCs are either immersed in fixative (paraformaldehyde for light microscopy or gluteradehyde for electron microscopy) or in RNA later or snap-frozen in isopentane-cooled in liquid nitrogen for molecular biological/biochemical analysis **(E)**. IHC, immunohistochemistry; IF, immunofluorescence; PLA, *in situ* proximity ligation assay; TUNEL, terminal transferase-mediated d-UTP nick end labeling; TEM, transmission eIectron microscopy; IGEM, immunogold EM.

Tissue for HOC (Figure 1) is transported to the laboratory as quickly as possible to minimize deterioration, ideally within minutes of collection. *“Time zero”* samples are processed concomitant with the onset of organ culture to establish morphological features prior to culture and treatments. Excess blood can be removed by immersing tissue in sterile phosphate-buffered saline prior to transfer into a sterile petri-dish for dissection into <1 mm^3^ fragments using two sterilized carbon steel single-edged razor blades (T585; Agar Scientific, UK) stuck together under a dissecting microscope (to aid in sample selection and orientation). However, other groups have documented use of various tools for dissecting tissue for OCs such as a Leica VT1200 S vibrating blade microtome with Vibrocheck (1), a biopsy punch (2, 3), motor-driven coring tool (4), and a Krumdieck tissue slicer (1, 5). For each tissue, multiple samples can be collected from different regions. For example, in the kidney samples can be taken from the medulla through to the cortex. The dissected fragments are immediately immersed in 200 µl of tissue culture medium (e.g., M199 containing 5% heat-inactivated fetal calf serum, 100 U/ml penicillin/streptomycin, 2.2 mM l-glutamine) in a sterile 96-well plate containing culture inserts, treated as indicated, or left untreated and maintained in a 37°C incubator (95% air, 5% CO_2_) for the desired time periods (usually less than 24 h). Culture conditions may need to be altered depending upon the tissue source, but we have successfully used this formulation for kidney, heart, and skin HOCs. Replicate samples for each treatment and time point are used to assess reproducibility. In our experience, we observe a rapid deterioration of morphology in culture of kidney samples incubated for longer than 18 h. During incubation, organ cultures remain metabolically and physiologically active and respond to external stimuli. Upon harvest, HOCs can be immersed in fixative (e.g., paraformaldehyde for light microscopy or gluteradehyde for electron microscopy), or in RNA later, or snap-frozen in isopentane-cooled in liquid nitrogen for molecular biological/biochemical analysis. Organ cultures of human kidney, heart, skin, and tumors can all be conducted using similar technique (6–10).

## Methods That Can be Applied on HOCs

**Figure 2:**
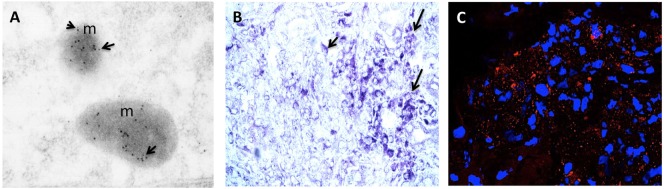
**(A)** RCC organ cultures treated with recombinant human TNF for 3 h and subjected to immunogold electron microscopy. Colloidal gold particles representing localization of phospho-MLKL (Ser358) (5 nm) and phospho-dyamin-related protein-1 (Drp1) (Ser616) (10 nm) and are detected in mitochondria (m). Mag: 105k×. **(B)** Representative light micrograph of RCC organ cultures treated with recombinant human TNF for 3 h were subjected to *in situ* hybridization using anti-sense probe to RIPK3 conjugated with Digoxigenin (DIG). Intense blue staining using anti-DIG-alkaline phosphatase and BCIP/NBT substrate (blue color) is seen in sites of gene expression within the cytoplasm of tumor cells. Original mag: 63×. **(C)** Representative confocal image of RCC organ cultures treated with recombinant human TNF for 3 h and subjected to *in situ* proximity assay to determine interaction between RIPK3 and phosphorylated-MLKL (Ser358). Numerous strong red fluorescent spots are evident within the cytoplasm of malignant TECs (mTECs) indicating close proximity of RIP3/pMLKL (<40 nm). Original mag: 63×. TNF, tumor necrosis factor.

Advances in molecular and biochemical techniques have increased the power of HOCs (Table 1). The ability to perform replicate cultures that can be sampled throughout a treatment period and the resolution offered by advances in labeling and imaging technologies allows detailed analysis of responses of both spatial and temporal to treatment within a tissue at a molecular level. Confocal microscopy can resolve molecules in the 200 nm ranges, and immunogold electron microscopy provides opportunities to resolve the cellular localization and phosphorylation status of proteins at a resolution of <10 nm (Figure 2A). Localization of gene expression can be studied using *in situ* hybridization (Figure 2B). New technologies can extend the potential of traditional immunolocalization assays to study molecular interactions. The *in situ* proximity ligation assay can be used to detect single molecules at a subcellular level and detect protein interactions in response to external stimuli (Figure 2C). The application of this technique to HOC provides the capability to study dynamic molecular interactions *in situ* with high spatial resolution.

**Table 1:** Methodologies that can be used to analyze human organ cultures and key findings.

Type of explants	Methodology	Key findings	Reference
Human skin	IHC	E-selectin is a marker for activation of endothelial cells (ECs)	(23)
Normal human skin and skin with psoriasis	Autoradiography, IHC, biochemical methods	[125] EGF binding was increased in psoriatic epidermis compared to normal skin, and increased EGF receptor phosphorylation	(26)
Newborn foreskins	TEM, IGEM, IHC	Microvascular endothelium of skin can undergo activation in response to exogenous/endogenous cytokines, with most pronounced changes seen at sites involved in leukocyte trafficking	(22)
Neonatal skin	IHC, WB	Cytokines known to be present in psoriatic skin induce EGF/TGF-α receptor and TGF-α expression in neonatal skin	(27)
New born human foreskin	IHC	Demonstrated mediators involved in the induction and regulation of ICAM-1	(15)
Human skin	IHC, IEM	Cytokine responses of microvasculature is altered in psoriasis	(31)
Human umbilical artery and vein	Gene delivery using adenovirus vectors in cultures	Demonstrated that adenovirus vectors are not of value for gene delivery to uninjured vascular endothelium *in situ*, and are unlikely to be suitable for genetic manipulation for vascular endothelium in organs for transplantation	(41)
Human tonsillar and appendix	WB	Demonstrated protein expression of mediators involved in upregulation of endothelial VAP-1	(13)
Human saphenous vein (de-endothelialized)	Histochemical stains (van Giessen, Miller’s elastic), H&E, IHC	ANG II increased DNA synthesis to a greater extent than in isolated cell cultures of saphenous vein smooth muscle cells, mediated in part by angiotensin receptor 1	(49)
Human skin	WB, RT-qPCR, geletainolytic zymogram	Demonstrated multiple cytokines can regulate expression of MMP-9 transcript and protein and in human skin	(18)
Human saphenous vein	MTT, Hoechst 333258, TUNEL, H&E, Verhoeff’s Van Gieson	Early molecular events occurring in the arterialization of human vein grafts are controlled by hemodynamic conditions	(67)
Normal cervical tissue and CIN II/III lesions	IHC	rhTRAIL and MG132, a proteosomal inhibitor, synergize to induce apoptosis, especially in CIN II/III lesions	(2)
Human umbilical tissue	RT-qPCR, IHC, FACS, WB	Arterial and venous ECs in cultured human umbilical tissue activate different transcription factors and upregulate different adhesion molecules in response to TNF	(24)
First trimester human placental villous and extravillous tissue	siRNA, WB	Silencing of GCM1 upregulated by forskolin treatment resulted in cell proliferation and promoted *de novo* syncitiotrophoblast formation in syncytially denuded floating villous explants	(46)
Human skin adhered to acellular dermis	RT-qPCR, IHC, histometric analyses, WB	HRG-activated HER3 contributes to the outgrowth process of epidermis	(30)
Healthy skin and skin from patients with ESRD	IHC, WB, ELISA	Production of MMP1, TIMP-1 but not type I procollagen is increased in skin of ESRD-treated with omniscan (gadodiamide). Indicated omniscan alters enzyme/inhibitory system responsible for collagen turnover in the skin and directly stimulates hyaluronan production	(36)
Normal human kidney, and renal transplants undergoing ACR and ATN	IHC, ISH, TUNEL	TNFRs are differentially regulated and activate different signaling pathways in normal, inflamed and ischemic human kidney	(9)
Human tonsil	Histology, IHC, and histochemistry	Reported preservation of structure and function in normal and neoplastic colon tissue	(39)
Human skin	IHC, FACS	Lentiviral tropism in skin tissue is distinct from tropism to keratinocytes in culture, and dependent on three-dimensional architecture of the tissue	(42)
Human neonatal foreskin	DNA extraction/Dot blot by image density quantification, IHC	Detected UV-induced DNA damage and repair in skin	(43)
Normal human kidney and RCC explants	IHC	Resident cancer stem cells are increased in RCC. Selective ligation of TNF receptor 2 can induce stem cell proliferation, increasing susceptibility to cell cycle-dependent cytotoxicity	(10)
Human saphenous vein and coronary artery	H&E, histochemical stains (Alcian blue, Miller’s elastin, and Van Gieson) Chemokine multiplex immunoassay, IHC, WB, histology, saturation and competition analysis, RT-qPCR	Quantified cell numbers and neointimal area in vascular cultures CCR-5 mRNA and protein in venous smooth muscle consistent with receptor binding, and that CCL4 and CCL5 are vasoconstrictors in the human saphenous vein	(20)
Human prostate cancer	Adenoviral gene delivery, IHC	STAT5a/b induces epithelial-to-mesenchymal transition in human prostate cancer	(48)
Normal human kidney and renal clear cell carcinoma (RCC)	IHC, TUNEL, IGEM, *In situ* PLA	Expression of TNFRs and signaling pathways activated are different in RCC compared to normal kidney	(8, 10)
Human colon and pulmonary biopsies	siRNA, WB	Silencing of Cyclin D1 expression by siRNA delivered by invivofectamine or nanoliposomes in colon but not lung organ cultures	(47)
Precision-cut human kidney slices	CytoTox-ONE homogeneous membrane integrity assay HPLC WB, RT-qPCR	Measurement of ATP content and LDH leakage indicated viability of cultures Measurement of uridine 5 diphospho-glucuronosyl transferase indicated cell functionality in cultures Determined TGFβ1-induced activation and upregulation of fibrotic markers at the RNA and protein level	(3)

*ESRD, end-stage renal disease; GCM1, glial cell missing-1; H&E, hematoxylin and eosin; IHC, immunohistochemistry; WB, western blot; RT-qPCR, real-time quantitative polymerase chain reaction; FACS, fluorescence activated cell sorting; siRNA, small interference ribonucleic acid; ELISA, enzyme-linked immunosorbent assay; HPLC, high performance liquid chromatography; TGF-α, transforming growth factor alpha; TGF, transforming growth factor; ATP, adenosine triphosphate; LDH, acetate dehydrogenase; VAP-1, vascular adhesion protein-1; rhTRAIL, recombinant human tumor necrosis factor (TNF)-apoptotic inducting ligand; TNFRs, TNF receptors; GCM1, glial cells missing homolog 1; HRG, high heregulin; EGF, epidermal growth factor; RCC, renal clear cell carcinoma; ACR, acute cellular rejection; ATN, acute tubular necrosis; NK, normal kidney; ICAM-1, intracellular adhesion molecule; TUNEL, terminal transferase-mediated d-UTP nick end labeling; LPS, lipopolysaccharide; ANG II, angiotensin II; CIN, cervical intraepithelial neoplasia; PLA, proximity ligation assay; IGEM, immunogold electron microscopy; TEM, transmission electron microscopy; STAT, signal transducer and activator of transcription; CCR-5, C–C chemokine receptor type 5; CCL, chemokine (C–C motif) ligand; VSMC, vascular smooth muscle cell; MMP, matrix metalloproteinase; TIMP, tissue inhibitors of metalloproteinase; HER, human epidermal growth factor*.

## HOCs Preserve Cellular Responses That may be Lost in Cell Cultures

Human organ cultures can be used to study physiological responses to mediators that are lost in cell cultures. For example, in organ cultures of normal human kidney, TGF-β1 promotes fibrogenesis in parallel with increased expression of multiple fibrotic markers including heat shock protein 4, fibronectin 1, type 1A collagen (COL1A1), and α-smooth muscle actin (α-SMA), but not vimentin, E-cadherin, and CD31. TGF-β1 pathway also increases plasminogen activator inhibitor 1, a downstream signaling molecule (3). These various responses to TGF-β1 differ from those seen in primary cultures or cell lines derived from different renal cell types (11, 12). In human tonsil organ cultures, pro-inflammatory cytokines, such as TNF, IL-1, IL-4, IFN-γ, and lipopolysaccharide (LPS) each induce marked upregulation of vascular adhesion protein (VAP)-1, a molecule important for leukocyte recruitment across high endothelial venules of secondary lymphoid organs (13, 14). Cultured endothelial cells (ECs), including ECs cultured from human tonsil, fail to express VAP-1 when exposed to these same agents. In human skin organ cultures, IFN-γ induces lCAM-1 expression primarily on ECs and epidermal keratinocytes (KCs) whereas IL-6, GM-CSF and macrophage-CSF induce ICAM-1 expression on both ECs and dermal dendritic cells (DCs) but not KCs. IL-1α or IL-β induce ICAM-1 expression on DCs while TNF affects primarily ECs and IL-3 does not affect any cell type (15). These varied responses to cytokines again differ from those of human dermal ECs and KCs in cell culture (16). In the same HOC system, the expression of the major histocompatibility antigens (MHC) class II (HLA-DR/DQ) is induced by IFN-γ in microvascular ECs and epidermal DCs [Langerhans cells (LC)] but not on KCs whereas IFN-γ does induce MHC class II molecules on cultured human KCs (17). Also in skin organ cultures, pro-MMP-9 expression is induced by TGFβ, which is increased by TNF (18). TNF (but not TGFβ) promotes proteolytic activation of pro-MMP-9 by conversion of the 92-kDa pro-MMP-9 to the 82-kDa active enzyme. This TNF-mediated proteolytic activation of pro-MMP-9 is undetected in isolated cultures of dermal fibroblast or epidermal KCs (19). In HOCs of saphenous vein both contractile and proliferative vascular smooth muscle cells (VSMCs) express CCR-5, a chemokine receptor implicated on atherosclerosis, in response to ligands CCL4 and CCL5 (20). In contrast, cultured VSMCs from human saphenous vein do not express CCR-5 when similarly stimulated (21).

## HOCs Can Reveal Positional Effects upon Cellular Responses

Endothelial cells play a key role in many different disease processes and while they are often considered as a single cell type, their phenotype, functions, and responses to stimuli *in vivo* are very sensitive to anatomic position. For example, the superficial vascular plexus (SVP) of human skin allows ready distinction of arterioles, capillaries, and venules with capillary arcades confined to the dermal papillae that nurture the epidermis and with paired arterioles and venules running parallel to the epidermal layer but below the epidermal rete. In response to TNF or LPS, only ECs lining postcapillary venules (PCV) of the SVP will express E-selectin (22), i.e., the same vessel segments typically associated with inflammatory infiltrates *in vivo* in elicited delayed-type hypersensitivity reactions (23). In the same HOCs, ECs lining arterioles and capillaries are responsive to these same mediators by increasing their expression of ICAM-1. In other words, the anatomic position of the ECs determines how, but not whether the ECs respond to specific inflammatory stimuli. In contrast, all cultured human dermal ECs will upregulate E-selectin and ICAM-1. Similarly, in HOCs of umbilical cords, where the paired arteries and single vein are also readily distinguished, the ECs lining umbilical veins respond to TNF by expressing E-selectin, whereas ECs lining umbilical arteries will not. In the same HOCs, arterial ECs respond to TNF by expressing higher levels of ICAM-1 than do umbilical vein ECs. Remarkably, these differences begin to narrow in as little as a few hours and are completely erased by 3 days in cell culture, at which time both arterial and venous ECs assume a common phenotype that differs from both types of ECs *in situ* and show intermediate degrees of both ICAM-1 and E-selectin induction. The change in the behavior of arterial ECs upon harvest and cell culture has been attributed to loss of shear stress-induced modifiers of the TNF response such as Kruppel-like factor 2, but the changes in venous EC, which also show loss of shear stress-induced factors, albeit from lower starting levels, are in the opposite direction of those observed in arterial ECs (24). The rapidity of the alterations in cytokine responses upon isolation supports the interpretation that the differences in TNF responses are determined by the microenvironmental context in which these cells are located, for example on distinct types of basement membrane, rather than by stable epigenetic changes. Thus, even though cell culture experiments allow the discovery and evaluation of cytokine actions on ECs, cultured ECs lose the ability to identify responses that vary with the anatomic origin of the cell, a feature amenable to study in HOCs. This loss of positional responses in human umbilical veins ECs occurs in both 2D and 3D cultures. Remarkably, when Bcl-2 transduced human umbilical vein ECs, which are indistinguishable from control ECs in 2D culture, are placed into 3D cultures and then implanted into immunodeficient mice, they form vessels that become perfused and acquire arteriolar, venous, and capillary morphologies and gain characteristic segmental responses to TNF. To date, organoid cultures that form vessels do not allow distinctions among vascular segment types.

## HOCs Can be Used to Study Stem Cells

Human organ cultures can be used to study stem cells within their *niche*. For example, in human cardiac tissue affected by ischemic heart disease, resident stem cells, identified as small round cells that express CD133 but not CD45, express TNF receptor 2 (TNFR2) and respond to TNF, in a manner dependent upon TNFR2 signaling, by entering cell cycle and expressing characteristic markers of mature cardiomyocytes (7). This potentially beneficial response of resident cardiac stem cells to TNF may have contributed to the adverse effects of anti-TNF therapies in human heart failure. HOCs can also allow comparisons of normal and tumor stem cells. Renal organ cultures also contain CD133^+^ resident stem cells, which are more abundant in renal clear cell carcinoma (RCC) than in normal kidney. In organ cultures of RCC and corresponding non-tumor kidney, TNFR2 engagement on CD133^+^ resident stem cells results in increased TNFR2 expression and promotes cell cycle entry. Importantly, while TNFR2 effects are not directly harmful to these stem cells, TNFR2-driven cell cycle entry increases their sensitivity to cell cycle-dependent cytotoxic agents (10). These findings have indicated that priming tumor stem cells with a TNFR2 agonist may enhance the efficiency of chemotherapy for RCC.

## HOCs Can Reveal Altered Responses and Signaling Pathways in Disease

### Inflammatory Diseases

Human organ cultures of skin from patients have provided insights into inflammatory disease pathogenesis. Psoriasis vulgaris, which displays hyperproliferation of the epidermal KCs, is characterized by overexpression and altered distribution of the EGF/TGF-α receptor, and of TGF-α itself. In normal stratified epithelium, the expression of EGF/TGF-α receptor is restricted to the basal and intermediate suprabasal KCs (25). However, in active psoriatic lesions, expression of EGF/TGF-α receptor is seen in the basal and subcorneal layers of stratified epithelium (26). TNF and IFN-γ induce EGF/TGF-α receptor expression in basal and suprabasal cells in non-stimulated explants in KCs in all layers of the epidermis (27). In contrast primary cultures of KCs cannot reproduce these position-related differences (28, 29). Moreover, treatment of skin HOCs with EGFR family members increases keratinocyte proliferation and neoepidermal thickness (30), mimicking psoriasis-like changes. ECs of the SVP in skin HOCs generated from psoriatic lesions, sites uninvolved by lesions and perilesional skin from the same patient provide an additional example of disease-related altered responses. The ECs in cultures from uninvolved sites and perilesional sites do not show expression of E-selectin and VCAM-1, whereas in lesional skin, both molecules are expressed on ECs of PCV and, significantly, on the ECs lining the remodeled (venularized) capillaries in the dermal papillae of the SVP. In skin HOCs from uninvolved sites, similar to skin HOCs from healthy donors, TNF induces E-selectin on the ECs lining PCVs whereas VCAM-1 is not inducible on these same cells, even when TNF is combined with IL-4. However, in perilesional skin biopsies from psoriatic patients, which is devoid of leukocytes, VCAM-1 can be induced on SVP venular ECs by the combination of TNF and IL-4, suggesting a change in the responsiveness of these ECs related to a disease process that precedes overt plaque spreading (31).

Human organ cultures of normal and pathologic colon have demonstrated that different bacterial species adhere in a peculiar manner to the proximal and distal colonic compartments of the mucosal surface (32). A consistent adhesion of *Streptococcus thermophillus* and *Bifidobacterium infantis* but not *Lactobacillus acidophilus*, with *B. infantis* adhering to the proximal compared to the distal segments. In contrast to cell cultures of human intestinal epithelial cell lines, which show differential adhesion profiles of bacterial strains (33), with *L. acidophilus* as the most adherent strain in intestinal mucosa (34). Findings from cell cultures for bacterial adherence capacity to epithelial cells are difficult to extend to the situation in the human gastrointestinal tract and it is unknown if intestinal organoids will be more useful in this regard.

### Systemic Diseases

Human organ cultures of skin have been used to study how systemic processes may influence cutaneous responses. Magnetic resonance imaging gadolinium-based contrast agents (GBCAs) appear to be a trigger of nephrogenic systemic fibrosis in a small subset of patients with end-stage renal disease (ESRD) (35). DaSilva et al. (36) compared responses to GBCAs in human skin organ cultures and in isolated dermal fibroblast and KC cultures, using skin from individuals with ESRD and from normal individuals. Skin organ cultures treated with GBCA (Omniscan) show increased MMP1 production, TIMP-1 and hyaluronan production but not collagen type-1. Responses were seen in patients with ESRD and controls, but basal levels were higher in patients with ESRD. These potentially pathogenic responses were detected in KCs in monolayer culture, and although similar responses were observed in fibroblasts, there was no difference in responses in 2D cultured fibroblasts isolated from the skin of normal patients as compared to patients with ESRD.

### Organ Transplantation

Cytokine effects in human kidney are cell type specific and also depend on anatomical location. In normal kidney, ECs of glomerular and peritubular capillaries (PTCs) express higher levels of TNF receptor 1 (TNFR1) associated with inactive apoptosis signal-regulating kinase-1 (ASK1p^Ser967^) and an absence of activated ASK1 (ASK1p^Thr845^). Other renal cell types lack both TNFR1 and ASK1. TNFR2 is only minimally expressed in any cell type in normal kidney. This expression pattern of TNFRs differentiates renal cells *in situ* from cultured renal cells where both receptors are typically expressed in the same cell. It also differs from the patterns seen in rejecting renal allografts or ischemia reperfusion injury where resident renal cells lose TNFR1 expression and ECs of PTCs and tubular epithelial cells (TECs) express TNFR2 (9). Effects of TNF signaling on different renal cell populations can be analyzed in organ cultures. Treatment of normal kidney organ cultures with a TNFR1-selective mutein results in activation of ASK1 and induction of cell death in ECs of glomerular and PTC, while a TNFR2-selective mutein upregulates TNFR2, results in phosphorylation of endothelial-epithelial tyrosine kinase (Etk) and initiates cell cycle entry in ECs of PTC and in TECs. TNF does not affect the expression of either receptor in cultured renal cells. In kidneys injured by allograft rejection, despite showing reduced or even absent expression of TNFR1, activated ASK1p^Thr845^ is seen in both ECs and TECs, and this has been linked to expression of an alternative receptor, DR3, that is absent from cultured renal cells (9).

### Cancer

In RCC organ cultures, a TNFR1-selective mutein promotes cell death in malignant TECs (mTECs) selectively expressing ASK1, with the majority of the cells dying *via* necroptosis involve the receptor-interacting protein (RIP1)/RIP3/phosphorylated mixed lineage kinase domain like (MLKL)/dyamin-related protein-1 (Drp1). As an example of how modern morphological approaches can be applied in HOCs, physical interactions between signaling intermediates have been demonstrated using *in situ* proximity ligation assay. In the same HOC system a TNFR2-selective mutein promotes cell cycle *via* reciprocal phosphorylation of Etk and vascular growth factor receptor 2 (VEGFR2) in mTECs, whereas VEGF activates VEGFR2, but not Etk, suggesting Etk–VEGFR2 interactions and cell cycle activation in mTECs are specific to TNFR2 signaling. TNFR2 expression increases with increasing cancer grade providing a model to study the effects of regulated expression of the receptor in a pathophysiological setting. These data have important therapeutic implications, suggesting that inhibitors of VEGFR may be more effective than neutralization of VEGF because TNF-TNFR2 signaling may provide an alternative and possibly more effective VEGF-independent means to activate this mitogenic receptor (8). Once again, this behavior revealed in organ culture cannot be replicated in isolated cell cultures of human TEC lines (DPK-KTEC-H and HK2). In the context of brain tumors, tumor cells’ susceptibility to TNF-related apoptosis-inducing ligand (TRAIL) and lack of activity in healthy cells led to speculation that TRAIL could be used in cancer therapy (37). However, in organ cultures of adult human brain slices, trimerized TRAIL increased death in a large number of cells in the cortex and white matter as compared to untreated cultures with damaged cells identified as neurons, oligodendrocytes, astrocytes, and microglial cells (38). Another study examined the role of calcium (Ca^2+^)/calcium-sensing receptor (CaSR) in regulation of growth and differentiation in normal and malignant colon HOCs (39). Lower third of the crypt in normal cultures showed proliferation while extracellular CaSR expression was present in the surface epithelium and upper third. E-cadherin and β-catenin were expressed at the cell surface between adjacent cells. In malignant tissue, in contrast to normal tissue, proliferation was detected throughout the epithelium with diffuse or absence of CaSR. Expression of β-catenin was detected throughout the cytoplasm and nucleus. Expression of E-cadherin was seen at the cell surface (as in the normal tissue) with diffuse expression throughout the cytoplasm in some areas. In contrast to colon carcinoma cells where extracellular Ca^2+^ did not induce E-cadherin or result in a shift in β-catenin from the cytoplasm to the cell membrane, Ca^2+^ treatment resulted in growth reduction in mock-transfected cells and in a Ca^2+^-responsive variant line derived from the same parental colon carcinoma cells. This was accompanied by increased production of E-cadherin and a shift in β-catenin distribution from the cytoplasm to the cell membrane (40).

## HOCs Can be Used to Study Therapeutics

Human organ cultures of blood vessels have been used to determine gene delivery efficiency of a replication-defective adenovirus 5 vector carrying the [beta]-galactosidase reporter gene to vascular ECs (41). In contrast to cultured human primary vascular ECs, which were efficiently infected (>90%) at adenovirus concentrations of >10^10^pfu/ml, non-dividing vascular endothelium *in situ* was very poorly transduced, casting doubt on the utility of this class of vector *in vivo*. Similarly, skin HOCs have been used to characterize the factors that determine lentiviral vector tropism and findings compared to monolayer cultures of KCs (42). While early monolayer cultures of progenitor KCs expressing keratin 15^+^/63^+^ are lentiviral vector-permissive, they are resistant to transduction in their native *niche* in the skin. Alternatively, transiently amplified keratin 14^+^ KCs are permissive to lentiviral transduction, in cell culture and in the skin, after separation of epidermis from dermis layer. Keratin 14^+^ KCs in the human skin hair follicles are resistant to lentiviral transduction even after partial digestion of the extracellular matrix (ECM) collagen. Thus lentiviral vector tropism to KCs in organ culture is distinct from tropism to KCs in cell culture.

In HOCs of skin, topical imiquimod, a pharmacological agent that facilitate the development of skin cancer through accumulation of genetic lesions failed to enhance DNA repair after UV-exposure (43). In contrast to imiquimod-treated epidermal bone marrow-derived cells (BMDCs) (43), attributing these differences to the KCs in the epidermal layer lacking TLR7/8 while BMDCs such as LCs express TLR7/8 (44). A differential response to imiquimod has also been observed in stimulated KCs monolayers (HaCaT cells, immortalized human KCs and primary KCs), which failed to show DNA repair in contrast to stimulated KG-1, a monocytic cell line (43). These data suggested that LCs activated by imiquimod might potentially influence neighboring KCs and thus provide an indirect protection against UV damage. HOCs of normal squamous cervical tissue and CIN II/III lesions have been used to evaluate the effect of a potential anticancer drug rhTRAIL (recombinant human TRAIL) alone or in combination with a proteasome inhibitor MG132 (2). MG132 induced increased sensitivity in CIN II/III cultures as compared to normal cultures, which was enhanced by treatment with rhTRAIL. In contrast, immortalized and transformed cells lines, which serve as a model for cervical carcinogenesis, show variable sensitivity to proteosomal inhibition to rhTRAIL (45). Among the human cervical cancer cell lines HeLa were moderately, CaSki was highly and SiHa was insensitive to rhTRAIL-induced apoptosis. However, MG132 resulted in a marked rhTRAIL-induced cell death in HeLa and SiHa in a time- and caspase-dependent manner. HOCs can also be treated with various pharmacological inhibitors, short interfering RNAs (siRNA), or anti-sense oligonucleotides to gain insights into molecular function of cells *in situ* (46–48). In human colon and lung organ cultures, stimulus LPS-activated endothelium (EC-LPS), induced a marked expression of Cyclin D1. Treatment of cultures with siRNA for Cyclin D1, in presence or absence of EC-LPS, resulted in approximately 46% reduction of Cyclin D1 compared to the basal condition, and by approximately 65% compared to colon samples treated with EC-LPS after 24 h (47). In organ cultures of prostate cancer, in which Stat5a/b-mediates epithelial-to-mesenchymal transition (EMT) (epithelial-mesenchyme transition), disruption of Jak2/Stat5a/b signaling by specific inhibitors resulted in a significant decrease in active nuclear Stat5a/b but not Stat3, increase levels in E-cadherin and simultaneously a decrease in levels of Twist1 indicating that active Stat5a/b signaling promotes expression of EMT markers (48). In studies of first trimester human placental explants denuded of villous cytotrophoblasts, siRNA and anti-sense oligonucleotides silencing of GCM1 (glial cell missing-1 transcription factor), which mediates syncytialization and proliferation, resulted in a strong inhibition of syncytiotrophoblast reformation, and increased proliferation of cytotrophoblast (46). In organ cultures of human de-endothelialized saphenous veins, an effector peptide of the renin-angiotensin system Angiotensin II (Ang II), induced a strong increase in DNA synthesis over 7 days, associated with increased proliferation and hyperplasia in media of vein. Treatment of cultures with Tyrphostin-23; a selective inhibitor of tyrosine kinases significantly abolished the effects of Ang II while PD123319, a selective AT_2_ receptor antagonist had no significant effect on the Ang II response (49). In contrast Ang II failed to stimulate DNA synthesis in cultured SMCs from human saphenous vein or from human coronary arteries (50, 51).

## Comparison of HOCs to Emerging Cell Culture Methods

### 3D Cultures

A wide range of 3D *in vitro* models is emerging to better mimic the human physiology and, at the same time, to reduce animal experiments (52). 3D cultures use different types of porous scaffolds, derived from naturally and engineered substrates (53). Gene expression profiles of cancer cells often differ in cells grown in 3D as compared to 2D cultures. In particular genes involved in angiogenesis, proliferation, invasion, migration, and chemosensitivity (54). 3D cultures have been used to determine the effects of cytokines (55) and cells seeded into a 3D cardiac ECM scaffold have shown increased calcium signaling and kinetics promoting stem cell maturation (56). However, in contrast to HOCs, ECM scaffolds used in 3D cultures fail to emulate the natural biochemical and physical properties of the ECM recognized as independent factors that influence cell activity (57). Furthermore, basement membrane-derived scaffolds may contain undesired components like viruses and growth factors. Indeed, other matrices permit cell attachment but not easy detachment of cell making assay development difficult. Even with the generation of 3D biological scaffolds *via* organ de-cellularization (58), current systems fail to offer complex *in vivo* tissue vasculature for supply of nutrients and oxygenation as well as removal of waste material necessary for promoting attachment, differentiation, and proliferation of cells. Moreover, clonal variation across various strains of cell lines and 3D culture techniques used in different laboratories may also influence data. Unlike HOC, cell lines used in 3D cultures lose many of their native *in vivo* characteristics once removed from the primary tissue. Moreover, most research that uses *in vitro* models ignores the origins of cell lines and histological characteristics because of the unavailability of these data. For example, SKOV3, an epithelial ovarian cancer cell line widely used in 3D *in vitro* model for studying ovarian cancer bears striking histological resemblance to a clear cell ovarian cancer, which is the least common histological subtype of invasive disease (59).

### 3D Bioprinting

This system incorporates biology and tissue engineering to develop biological substrates that restore, improve, and maintain tissue function. The three most common 3D-bioprinting mechanisms include inkjet (a non-contact printing technology that reproduces digital patterns onto a substrate using tiny ink drops), laser (uses laser energy to vaporize the solution of biological samples and eject the remaining substances), or extrusion bioprinting (uses temperature-controlled polymerized materials for scaffold fabrication) (60). All these methods combine solid free-form fabrication and precise placement of cells and other biological factors to the desired 2D and 3D positions. This system offers additional biocompatibility and capacity for uniform cell incorporation. It begins with intrinsic organization and offers a template to guide reconstruction of cells and thus create a biomimmicked tissue with high throughput and cell manipulation. Use of personalized tissue substitutes promotes and enhances regeneration in areas of defected tissue (61). This system has shown promise in gene and drug delivery with precise placement during tissue construction. However, compared to HOCs, the limitations of this system include difficulties in managing single cells, over-drying leading to the failure of biological systems, and cell damage and altered phenotype caused by the printing process. Bioprinting requires fine-tuning of matrices for optimal conditions of simulation and deposition of cells and scaffolds. Also the current strategies still cannot fabricate new tissue that is indistinguishable from native tissue *in vivo* with respect to the zonal organization, ECM composition, and mechanical properties. Introduction of structures *in vivo* to facilitate organ regeneration have shown that infused cells may fail to maintain their formation in the recipient (61). Thus, the precise delivery of cells and biological factors to the desired 3D cultures is still far from being resolved.

### Organoids

This system stands at the forefront of 3D approaches as it more accurately recapitulates *in vivo* characteristics of the original individual’s tissue because the ECM and cell–cell interactions are intrinsic to the culture. Organoids have been successfully generated from human healthy and diseased tissue and pluripotent stem cells (62). Organoids from primary tissue sustain basement membrane extracts that are hallmarks of the original tissue in terms of architecture, cell type composition, and self-renewal properties. Patient-specific organoids have allowed functional genomic studies that can be related to the diagnosis of a patient and, with future development, will contribute to the generation of personalized therapies. However, in comparison to HOCs, organoids lack histological diversity and sequential differentiation as occurs in adult human tissue. They also lack surrounding tissue that is important for the interplay of cell–cell and cell–ECM cross talk, requires structural support to promote continuity and proper orientation of cell growth and can be quite heterogeneous from tissue to tissue and fail to generate tissue of origin. For example generation of cerebral organoids that have used embryonic bodies (EBs) kept in suspension leads to uniform neural ectoderm formation along the outer surface of EBs, whereas inner non-neural mesendodermal tissues do not develop, and the neurons do not display a six-layered architecture such as that seen *in vivo* (63). Cultures are also prone to acquiring independent secondary alterations and can quickly grow beyond the limits of stationary diffusion of oxygen and nutrients. Thus, although promising in their application, 3D cultures and organoids have not as yet accurately reflect the characteristics of primary human cells *in vivo*.

### Organ-in-a-Chip

This emerging 3D cell-culture model better mimics the microstructure, dynamic mechanical properties, and biochemical functionalities of living organs. It integrates microfluidic devices created with microfabrication techniques (photolithography, replica molding, and microcontact printing) using living cells cultured within 3D devices to study human physiology in an organ-specific context, and to develop specialized *in vitro* disease models (64). Structures with defined shapes and positions are created by microfabrication techniques on the micrometer scale to position cells and tissues, control cell shape, and function, and create highly structured 3D culture microenvironments. The development of a multi-analytic optical sensing module for dynamic measurements of pH and dissolved oxygen levels in the culture medium has been recently reported (65). Small amounts of fluids are manipulated in microfabricated hollow channels to generate and precisely tune dynamic fluid flows and spatiotemporal gradients, as well as deliver nutrients and other chemical cues to cells in a controlled manner. However, HOCs retain several advantages over organ-on-a-chip. The latter still remains an artificial model relying on microfabricated scaffolds to mimic ECM, and thus failing to recapitulate *in vivo* tissue. These systems lack multi-organ interactions that are critical to some aspects of drug metabolism and toxicity. Interrelationship and cross talk between multiple cell types and the dynamics for modulating physiological processes are currently too complex to be recapitulated in such a system. Table 2 summarizes the strengths and weakness of all emerging cell cultures as compared to HOCs.

**Table 2:** Summary of the strength and weakness of the human organ culture (HOC) with emerging cell cultures.

	Strength	Weakness
2D cell cultures	Simple model, easy to manipulate, low cost	A static system of cell growth that does not reflect the situation *in vivo*. Lacks cell–cell and cell–extracellular matrix (ECM) interactions
	Uniform rich oxygenation. Nutrients are provided to all cells, and waste products are secreted directly into the media	Growth on plastic flasks/plates affect polarity, morphology and migratory properties of cells
	Easy environment to control, allowing manipulation of cells	Extended passages may alter the phenotype and histological characteristics of cells
	Can be used as a platform for drug testing	Fails to mimic a physiological microenvironment. Inability to depict traits exhibited using *in vivo* systems
	Can be conveniently observed and analyzed by imaging techniques	

Three-dimensional (3D) cell cultures	Recreates cell–cell and cell–ECM contact that mimic the *in vivo* natural environment promoting spatial organization and high density culturing	More expensive and time consuming than 2D cultures
	Provides a simple but efficient tool for investigating cellular responses non-invasively and in real time under physiologically relevant conditions	Lacks natural stromal architecture and some molecules important for promoting attachment, differentiation and proliferation of cells
	Cells can survive prolonged incubation	ECM scaffolds do not recapitulate the complexity of the *in vivo* matrix and substrate elasticity can effect cell morphology and function
	Great promise for drug discovery, provides predictive response of drug activation and safety in organ-specific cells	ECM scaffolds may contain biological pathogens and show batch-to-batch variability in composition
	Show better response to exogenous stimuli and withstands more stress when countered by cytotoxic agents	ECM scaffolds may effect mechanical strain and flow shear stress dynamic models
	Induces changes more relevant to disease models	Vascular networks lack segmental structure
		Cell morphology/function may alter in culture, either as a result of passaging before introduction into the scaffold or direct contact with the scaffold

3D Bioprinting	Provides material with optimal rheological properties that can restore, maintain, and improve tissue formation	Very expensive and technically difficult. Requires fine-tuning for optimal conditions. Different imprinting methods have different limitations
	Offers additional biocompatibility and capacity for targeted cell incorporation at specific sites	Printing process may cause cell damage and alter cell phenotype. Infused cells may not be incorporated
	Potential for high throughput when optimal conditions have been established	Materials can suffer batch-to-batch variation and difficulties with scale-up

Organoids	Retains tissue identity and closely recapitulate 3D structural organization	Can require scaffolds/polymers to provide structural organization and promote continuity and proper orientation of cell growth
	Amenable to high throughput drug screening; patient-derived organoids allow 5personalized therapy design	Growth of cells requires careful formulation of media/supplements
	Can be readily expanded and frozen to create a master “cell-bank” for subsequent use	Cell growth can be limited by the diffusion of oxygen and nutrients into the organoids
	Ability to capture sub-clonal populations *in vitro*	Lacks secondary tissue that can be important for the interplay of cell–cell and cell–ECM cross talk
	Can generate tissue from pluripotent cells	Can display significant variability between preparations
	Can be maintained for more than 1 year in long-term culture	
	Serial examination allows developmental study of tissue	

Organ-on-a-chip	Multifluidic chambers allow for the possibility of tissue/organ interconnection	Scaffolds used as ECM fails to mimic *in vivo* architecture and lack interrelationship and cross talk from multiple cell types
	Utilizes smaller media volumes than static cultures in wells	Simplified ECM can lead to matrix degradation or contraction and may suffer a high batch-to-batch variation
	Fluid flow between channels allow prolonged culture periods than static cultures and facilitates cell growth environment	Complex channel designs make it difficult in cell seeding
	Multi-organ system allows assessment of drug efficacy and toxicity predictions	Relies on a superficial model system to deliver fluids *via* microfluidic chambers
	Allows high throughput sample processing and more realistic sample size	Variation in tools/scaffold/fluid quantity can affect outcome
	Transparent nature of the fabrication materials makes imaging simpler	Fabrication process can prove technically challenging, leading to induction of bubbles and flow perturbations that can destroy cell cultures
		Cell media may influence cell phenotype
		Prolonged cultures reduce tissue viability

HOCs	Provide cell–cell and cell–ECM interactions in a natural environment preserving endogenous molecules and growth factors	Human samples may be difficult to obtain; limitation in sample numbers may affect experimental design
	Preserve the naturally complex stromal architecture, vascular networks, and parenchymal anatomy facilitating better response to external signals	Tissue viability reduced by prolonged cultures
	Gene/protein expression profiling/imaging can be carried out on cultures using established tissue assays	Large samples may restrict delivery of oxygen and nutrients and penetration of exogenous stimuli
	Variability in patient-to-patient responses to exogenous stimuli can be assessed	Real-time monitoring may be limited by the depth of the thickness of the tissue

## Outstanding Questions

In this review, we have summarized how HOCs offer several significant advantages as compared to conventional and newer 3D cultures for studies of patho-physiologic mechanisms that better reflect the *in vivo* situation. However, HOCs also have some limitations. Chief among these is tissue acquisition, which results in limited sample size, variability, and heterogeneity. Heterogeneity and variability are problems faced in many human investigation and genetic analyses may help in stratification and lead to classifications into subtypes. In other words, this may be turned into an advantage if sufficient numbers of samples are available. Acquisition will remain a problem but can be partly addressed by an organized and efficient approach to ethical sample collection. Second major challenge is the limited viability of most organ culture systems. In our hands, deterioration in tissue architecture occurs after 18 h incubation. In general, more extended periods of survival will be needed to allow genetic techniques, such as RNAi-mediated gene knockdown or vector-mediated overexpression to be carried out successfully. Protein transfection using cell-permeating tags on wild-type, constitutively active, or dominant-negative proteins may be used as alternatives. Other problems include uneven penetration or cellular uptake of vectors. Experience with siRNA in organ cultures has shown some success (46–48, 66). A third current limitation of HOCs that needs to be addressed is the lack of perfusion leading to metabolic perturbations in the microenvironment. Perfusion itself may influence the system by providing shear stress and pulsations, physical signals especially important for vascular studies. Attempts have been made to address this issue (67), but further advances will be needed to develop a physiological flow system that allows for controlled hemodynamics in HOCs.

## Conclusion

In contrast to other experimental cultures commonly employed for basic research or preclinical drug testing in the human system, HOCs offer the advantage of studying tissue cells remaining within their at least partially intact microenvironment. Increasing data demonstrate that cell lines, when kept in culture too long, exhibit reduced or altered key functions and often no longer represent reliable models of their original source material. HOCs preserve the integrity of cells and matrices in an organ-specific structure and are more likely to be clinically relevant. The response of a whole integrated tissue to a simple ubiquitous signal will reflect not just the specificity of the signal or the receptor affinity, but also the microenvironment of juxtaposed cell types and ECM. Structural integrity is a major reason for adopting organ culture as an *in vitro* method instead of cell culture. HOCs can model a wide range of applications that permit many functional and mechanistic studies and provide a simple but informative model in which to study the effects of pharmacological agents, cytokines, and various other mediators in humans.

## Author Contributions

Outline of the review: RA-L and JP. Wrote the review: RA-L. Edited the text: JB and JP.

## Conflict of Interest Statement

The authors declare that the research was conducted in the absence of any commercial or financial relationships that could be construed as a potential conflict of interest.
